# A multi-user multi-robot multi-goal multi-device human-robot interaction manipulation benchmark

**DOI:** 10.3389/frobt.2025.1528754

**Published:** 2025-09-25

**Authors:** Akito Yoshida, Rousslan Fernand Julien Dossa, Marina Di Vincenzo, Shivakanth Sujit, Hannah Douglas, Kai Arulkumaran

**Affiliations:** Araya Inc., Tokyo, Japan

**Keywords:** shared autonomy, human-robot interaction, multi-agent, multimodal, benchmark

## Abstract

One weakness of human-robot interaction (HRI) research is the lack of reproducible results, due to the lack of standardised benchmarks. In this work we introduce a multi-user multi-robot multi-goal multi-device manipulation benchmark (M4Bench), a flexible HRI platform in which multiple users can direct either a single—or multiple—simulated robots to perform a multi-goal pick-and-place task. Our software exposes a web-based visual interface, with support for mouse, keyboard, gamepad, eye tracker and electromyograph/electroencephalograph (EMG/EEG) user inputs. It can be further extended using native browser libraries or WebSocket interfaces, allowing researchers to add support for their own devices. We also provide tracking for several HRI metrics, such as task completion and command selection time, enabling quantitative comparisons between different user interfaces and devices. We demonstrate the utility of our benchmark with a user study (n = 50) conducted to compare five different input devices, and also compare single-vs. multi-user control. In the pick-and-place task, we found that users performed worse when using the eye tracker + EMG device pair, as compared to mouse + keyboard or gamepad + gamepad, over four quantitative metrics (corrected p < 0.001). Our software is available at https://github.com/arayabrain/m4bench.

## 1 Introduction

Most human-robot interaction (HRI) research focuses on real robots and specific use-cases, but this can make reproducibility and comparisons between approaches difficult. In contrast, the artificial intelligence community places emphasis on benchmarks in order to track progress in algorithmic development. For instance, many continuous control algorithms are first tested on benchmark tasks in MuJoCo (Todorov et al., 2012), and later become deployed on real robots.

**FIGURE 1:**
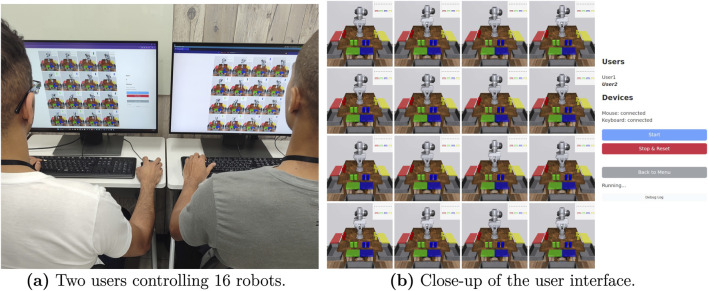
Usage of M4Bench. **(a)** Multiple users can join the same session for multi-robot control through our web-based server-client. **(b)** M4Bench supports controlling up to 16 robots simultaneously, with info, diagnostics and experimental controls available in the panel on the right.

Driven by this ethos, we developed a multi-agent (Dahiya et al., 2023), multimodal (Su et al., 2023) HRI benchmark in order to study the interaction between multiple users and multiple robots (Figure 1), as well as the usability of different input devices, in a shared autonomy paradigm. Whilst we have designed the overall structure of M4Bench to be modular and extensible, its architecture is particularly well-suited for investigating human-robot collaboration (HRC), in which humans and robots work together closely in a shared environment to achieve common goals through mutual interaction and coordination (Ajoudani et al., 2018; Villani et al., 2018; Nikolaidis et al., 2017), and particularly in situations involving shared control and physical manipulation. While other robot types—such as quadrupeds or drones—are also explored in HRC research, M4Bench currently focuses on robotic arm manipulators, given their widespread adoption and utility in accomplishing collaborative tasks that involve physical interaction.

Our goal was to make a flexible benchmark that scales across many dimensions: it supports multiple users, multiple robots, multiple goals (for each robot), and multiple input devices (M4Bench; Table 1). Unlike prior benchmarks that perform subsets of these comparisons (Saren et al., 2024), or that focus primarily on multi-robot systems (Puig et al., 2020; Zhang et al., 2023; Mandi et al., 2024; Esterwood and Robert Jr, 2023), human-robot coordination (Zhang et al., 2023; Thumm et al., 2024; Mandi et al., 2024), or planning and control of robots or embodied agents via natural language (Mandi et al., 2024; Chang et al., 2024), our benchmark enables investigation of the usability, scalability and ease-of-use of different input modalities under multi-user and multi-robot configurations, in a controlled and reproducible setting.

**TABLE 1:** Comparison of our benchmark with existing HRC benchmarks (Section 2.2) across four axes: multi-user (more than one user), multi-robot (more than one robot), multi-goal (tasks completed by choosing among discrete options; variable setting allows experimenter to change available sub-tasks), and multi-device (more than one input device).

Benchmark	Multi-user	Multi-robot	Multi-goal	Multi-device
Watch-and-Help (Puig et al., 2020)	F	F	F	-
Co-ELA (Zhang et al., 2023)	F	-	F	F
The Warehouse Robot Interaction Sim (Esterwood and Robert Jr, 2023)	F	V	F	F
Human-Robot Gym (Thumm et al., 2024)	F	F	F	F
RoCo (Mandi et al., 2024)	F	V	V	-
PARTNR (Chang et al., 2024)	F	F	V	F
M4Bench (Ours)	V	V	F	V

For each axis, we specify the adequate setting among the following three possibilities: F means that the benchmark supports this factor in a fixed configuration; V means that the benchmark supports varying this factor; and - indicates that the modality is either not available or not applicable.

Our implementation addresses several under-explored practical challenges in HRI studies. Firstly, supporting multiple simultaneous human users and robots to be controlled in a shared control loop requires robust session management. Secondly, we built a modular input device interface that not only integrates conventional inputs (e.g., keyboard, mouse), but also biosignal-based devices (e.g., eye trackers, wearable electrodes), thereby allowing researchers to systematically evaluate usability and cognitive load across control modalities. In the current setup for M4Bench, the task (pick-and-place) and robot controllers (inverse kinematics) were deliberately picked to be relatively simple, which allows us to achieve better consistency in quantifying HRI (Zimmerman et al., 2022). Finally, while various factors may be linked in other benchmarks, M4Bench allows independent and controlled variation across number of users, number of robots, number of goals, and input device combinations, making it possible to isolate key variables in shared autonomy studies.

We also conducted a user study (n = 50) to show the utility of our benchmark. In the user study we were able to test hypotheses over two different settings: a comparison over input devices, and a comparison of single-vs. multi-user control. We found significant differences between input devices, and largely no difference between differing numbers of users^1^. Our M4Bench software, available at https://github.com/arayabrain/m4bench, was designed to be extended, and we hope it will be of use to the HRI community.

## 2 Related work

### 2.1 Multi-agent HRI

Although most HRI studies involve a single human and single robot, HRI research has evolved to accommodate complex team dynamics that can include multiple users, robots, or both. A system’s team composition can be optimized to best suit the unique needs of the task environment at hand.

Multi-user, single-robot collaborative systems have been popular in coordinating search and rescue operations, where the robot is teleoperated in environments too hazardous for human users. In such cases, the collaborating users typically take on different roles. For example, one user may be operating virtual hands while the other monitors robot feedback (Szczurek et al., 2023).

Alternatively, the team could be composed of a single user and multiple robots. When multiple robots are involved in a system, it is important to consider whether they are homogeneous or heterogeneous. Teams with homogenous robots have been used in the context of human-swarm interaction (HSI) (Gale et al., 2018). Outside of HSI, operators have controlled homogenous robots to complete industrial workplace tasks in mixed reality (Kennel-Maushart et al., 2023). Homogeneous frameworks have also conceptually studied to help with the identification of hazardous sources in turbulent environments (Ristic et al., 2017). Heterogeneous systems have been similarly used in search and rescue, where a single user commands a heterogeneous team of robots based on their capabilities (Liu et al., 2015). Regardless of the robot team’s composition, the human generally takes on the supervisor role that assigns tasks to the multiple robots. However, such systems can put excessive mental workload on a user (Podevijn et al., 2016). Several solutions have been proposed to combat user mental fatigue, including simplifying the interaction task when the mental load detected is deemed unsustainable for the user (Villani et al., 2020; Rosenfeld et al., 2017).

Finally, several teams have attempted to build multi-user multi-robot systems. Although many potential applications are still being evaluated, there have been several notable studies that have provided information on the important factors that contribute to developing such systems. Multi-robot systems have also been designed for social applications. In the classroom, robots have been used as teaching aids, such as helping children learn handwriting (Hood et al., 2015), or helping middle school students learn about atmospheric science (Özgür et al., 2017). Industrial applications of multi-robot systems have also been explored, such as in automotive manufacturing, where robots contribute to increased efficiency and safety on the production line (Wang et al., 2023; Kennel-Maushart et al., 2023).

As HRI systems become increasingly complex and deployed in a wide range of applications, comparing systems has become increasingly difficult. To effectively evaluate how systems compare, we must agree on standardised metrics.

### 2.2 HRI metrics and benchmarks

Given the diversity of aspects involved in a human-robot interaction, defining metrics that can fully capture every aspect is a complex task. Even more challenging is to define metrics that are generalizable across different studies. Indeed, such metrics would be expected to fit various experimental setups, regardless of the task, the type of robot, the number of users, or the control device employed.

In the early years of HRI, researchers already used a variety of application-specific metrics that were often not directly comparable (Steinfeld et al., 2006). This was mainly due to the interdisciplinary nature of HRI, which created an inherently decentralized research paradigm (Zimmerman et al., 2022). This fragmentation hindered the development of unified frameworks and slowed progress in the field. The milestone work of the DARPA/NSF Interdisciplinary Study on Human-Robot Interaction (Rogers and Murphy, 2002) identified the critical need for standardized metrics in HRI, which Steinfeld et al. (2006) built upon by introduced a comprehensive set of metrics for HRI, offering structured guidelines for evaluating various aspects of HRI. While much progress has been made since then, HRI metrics still remain an active research area.

Metrics in HRI have adopted a specific configuration across the community, typically categorized based on which aspects of the interaction they measure or evaluate. A survey from 2013 identified forty-two distinct metrics, with seven measuring the human, six measuring the robot, and twenty nine measuring the overeall system (Murphy and Schreckenghost, 2013).

Metrics can be both explicit qualitative subjective evaluations or implicit quantitative measures. There are five primary methods of evaluation used for human studies in HRI: (1) self-assessments, (2) interviews, (3) behavioural measures, (4) psychophysiology measures, and (5) task performance metrics. As reported in (Bethel and Murphy, 2010) it seems essential to use three or more methods of evaluation to establish study validity. The use of a single method of measurement is not sufficient to accurately interpret the responses of participants to a robot with which they are interacting. Using more than one way ensures a comprehensive study with reliable and accurate results that can be validated.

Self-assessments are a primary evaluation method in HRI studies, where participants provide direct feedback on their interaction experiences, perceptions, and overall satisfaction with the robot or system. Therefore, the HRI community is increasingly adopting standardized tools such as the NASA task load index (TLX) (Hart, 1988) for workload assessment and the system usability scale (Brooke, 1996) for evaluating usability.

On the other hand, performance metrics are based on different aspects of the robotics system, such as accuracy, speed, reliability, robustness, adaptability, scalability, usability, safety, and cost. Depending on the type, domain, and purpose of the robotics system, some metrics may be more relevant than others (Russo, 2022). Across the most commonly used performance metrics in HRI, we identified several key measures: task completion time; error rate; success rate; efficiency; task accuracy; and interaction effort among others (Steinfeld et al., 2006; Olsen and Goodrich, 2003; Hoffman, 2019).

Given our goal of developing a system capable of adapting to different tasks, robots, users, and control interfaces, we selected the following metrics for implementation: task completion time, command selection time, and error rate, combined with the NASA TLX as a standardised tool for workload assessment. A detailed description of these can be found in Section 3.5.

While these metrics can be adapted to specific studies conducted through our platform, they remain primarily suited for comparisons within similar studies and configurations. This underscores the importance of further exploring standardized metrics in HRI. To tackle this challenge, HRI benchmarks play a crucial role in providing a structured framework for testing and evaluation, ensuring consistency and comparability across different subfields and task groups.

Establishing benchmarks that encompass the diverse range of HRI contexts remains a challenge. Nevertheless, several efforts have been made by the research community to unify testing standards within specific categories. One significant advancement has been the adoption of simulated environments for HRI benchmarking. One of the first notable platforms for multi-agent interactions in realistic environments was VirtualHome (Puig et al., 2018), designed to simulate rich home settings where agents interact with objects and each other. The authors later introduced a benchmark alongside this platform, with a structured evaluation protocol assessing AI agents on success rate, speed-up, and cumulative reward to test generalization and collaboration (Puig et al., 2020).

Similarly, The Warehouse Robot Interaction Sim is an open-source immersive platform that provides a flexible environment for evaluating cooperative human–robot interaction tasks. It features real-time simulation, customizable task scenarios, and adaptive robot behaviours, allowing for in-depth analysis of interaction dynamics and task modifications as needed (Esterwood and Robert Jr, 2023). Another noteworthy initiative is Human-Robot Gym (Thumm et al., 2024), which offers HRC benchmarks with diverse collaborative tasks, supports multiple robot systems, and facilitates comprehensive evaluation through predefined tasks and reproducible baselines. Similarly, Mandi et al. (2024) introduced RoCo, a benchmark with tasks geared toward evaluating the ability of large-language models (LLMs) to control and coordinate robot arms, with the possibility of having a human directly interacting with a robot arm in the real-world while communicating via natural language. To the best of our knowledge, PARTNR (Chang et al., 2024) represents the most comprehensive benchmarking framework currently available. It integrates multiple evaluation methodologies, supports a wide range of collaborative tasks, and offers the most extensive set of standardized HRI assessments, making it a significant reference point in the field.

However, as shown in Table 1, unlike PARTNR and other existing benchmarks, our M4Bench introduces major flexibility across multiple axes, allowing variable configurations for multi-user, multi-robot, and multi-device testing.

### 2.3 Multimodal HRI

Multimodal HRIs have commonly been implemented in settings with industrial robots, assistive mobile robots, robotic exoskeletons, or robotic prosthetics (Su et al., 2023). Given that humans naturally communicate through multiple modalities, using multiple input or output devices simultaneously can improve system usability, particularly for users with limited motor control. In elderly users, fusing multiple input modalities has been found to significantly increase human gesture recognition performance (Rodomagoulakis et al., 2016). Multimodal systems have also been found to benefit hemiplegic users, who showed enhanced engagement and improved movement prediction when combining biological signals like electromyographs (EMG) and electroencephalographs (EEG) during rehabilitation (Gui et al., 2017).

Early works explored integrating visual and audio input to make intuitive HRI systems (Goodrich and Schultz, 2008). Since then, the effectiveness of diverse combinations of input modalities has been tested including voice and facial expression (Alonso-Martin et al., 2013), speech and gesture (Rodomagoulakis et al., 2016; Strazdas et al., 2022), and facial expressions with EEG signals (Tan et al., 2021). Input modalities have been more recently extended to include haptic feedback and physiological sensing (Wang T. et al., 2024; D’Attanasio et al., 2024). With recent developments in LLMs, LLM-based robotic systems are showing promise in HRI by demonstrating their ability to adapt to multi-modal inputs when determining appropriate assistive actions (Wang C. et al., 2024; Zu et al., 2024).

Several studies have compared different input devices for HRI. Some examples include: PS3 gamepad versus PC keyboard (Adamides et al., 2017), mobile robot control with an app versus gamepad (Mallan et al., 2017), and robotic navigation with a keypad versus a Nintendo Wii controller (Guo and Sharlin, 2008). All studies reported significant differences between devices and highlight the importance of selecting appropriate input methods for optimized HRI performance.

While existing HRI benchmarks (Table 1) typically focus on agent coordination, language grounding, or simulated avatar control, M4Bench was designed to foreground the human–robot interactions themselves—specifically in how they scale across different users, robots, goals and input modalities. This introduces several design and engineering challenges, including synchronized multi-user interactions and hardware abstraction for non-traditional control devices. M4Bench offers a foundation for systematically studying shared autonomy across these dimensions, as highlighted in Table 1.

## 3 Materials and methods

**FIGURE 2:**
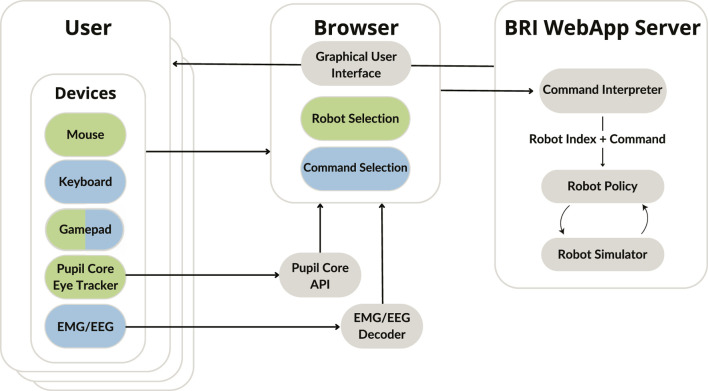
Software diagram. The brain-robot interface (BRI) web application server provides an endpoint to access the user interface, and additionally runs the underlying robot simulator, listening for user commands, and executing them. Users access the interface through a browser, which contains camera feeds from the robot simulator and experiment controls. The browser receives and processes user inputs either through native browser events (e.g., for mouse, keyboard, or gamepad) or dedicated processing modules (e.g., for an eye tracker or EMG/EEG electrodes). Device inputs map to *robot selection* and/or *command selection*.

Figure 2 provides an overview of our software, which consists of a web server (running robot simulators), a web interface that receives inputs and displays the robot(s), and, optionally, additional processes to translate inputs from devices such as eye trackers or EMG/EEG. The front-end uses standard HTML, CSS and JS, and is compatible with major browsers (Edge, Safari, Chrome and Firefox). The back-end uses Python and is compatible with Windows, OS X, and Linux. These software architecture choices were made to maximise compatibility and ease of extending the benchmark.

We built our web server using the FastAPI framework^2^. The web server responds to various HTTP endpoints, runs a multiprocess environment runner, manages WebRTC streams (delivering images from the simulators), and handles user session management. We built the front-end using Bootstrap^3^ to structure and manage the user interface. As shown in Figure 2, the web server, web interface and device processes use WebSockets to communicate real-time information, such as device outputs. A notable exception is the use of WebRTC for streaming video, as it is better suited for such use-cases, and can use peer-to-peer technology to send the images directly from the simulator to the interface.

The environment runner runs one robot simulator process per robot, allowing us to achieve real-time interaction even when scaling up the number of robots to control. We built a pick-and-place environment for our benchmark in RoboHive (Kumar et al., 2023), a robot learning framework that uses MuJoCo (Todorov et al., 2012) as its underlying robot simulator. A primary benefit of RoboHive is that it abstracts robot control policies for both real robots and simulated robots to have the same structure, making it easier to develop in a simulator and deploy in the real world: with the right configuration sets, deploying a controller tested in simulation on the real robot is simply a matter of changing an environment flag in RoboHive. Similarly, simulated sensors such as cameras can be replaced by their real-world counterparts by editing the environment configuration files. The other necessity for transferring planning-based manipulation controllers to the real world is object detection, which can be achieved either through ArUco markers (Garrido-Jurado et al., 2014) or other machine learning/computer vision methods (Bai et al., 2020).

Our benchmark is set up to allow users to control 1, 4, or 16 robots simultaneously. This allows us to display robots in a regular grid, which simplifies the layout for users. The different numbers of robots allow us to test how human-robot interaction scales across a range of robot numbers, with the single robot scenario also providing a simplified setting with which different input devices can be tested. Simultaneously, multiple users can join an experiment to control robots together by joining from a web browser. It is even possible to allow remote participation, if the web server is made accessible publicly.

### 3.1 Robot task and control

We constructed a simple pick-and-place task for HRI experiments, as our purpose is to test and quantify the interaction between humans and robots, and not the performance of robots at fulfilling complicated tasks. For the task, a robot arm—a 7 DoF Franka Panda—is placed on the centre of a large square table, with different groups of colored blocks to its sides and front, with bins for each group of blocks placed at the edge of the table. When the robot is instructed to pick a block of the specified color, it will begin picking the specified type of block and placing them in the corresponding bin one by one. We use four groups of two blocks each, which we found provided a good trade-off between goal diversity (number of groups) and robot execution time (amount of time spent picking and placing blocks).

In our task setting, the robot will ignore any other commands until all of the specified blocks are placed. Based on user feedback in early experiments, we added a LED indicator around the base of each robot which lights up when it is active, and remains off when it can be controlled again. As demonstrated in prior work (Baraka et al., 2016; Pörtner et al., 2018), light indicators are cheap and effective tools for HRI.

The robots are controlled through a simple inverse kinematics motion planner with hard-coded waypoints to place the end-effector above a block, reach down and grasp it, and move it above the bin before opening the gripper. Once the path is planned, the trajectory is executed as fast as possible whilst respecting joint velocity limits. Although this planner does not guarantee 100% task success, in practice we never observed a single failure. However, in order to ensure that experiments can always be completed, if the planner were to fail our software will still count it as a success for the user.

### 3.2 User interface

**FIGURE 3:**
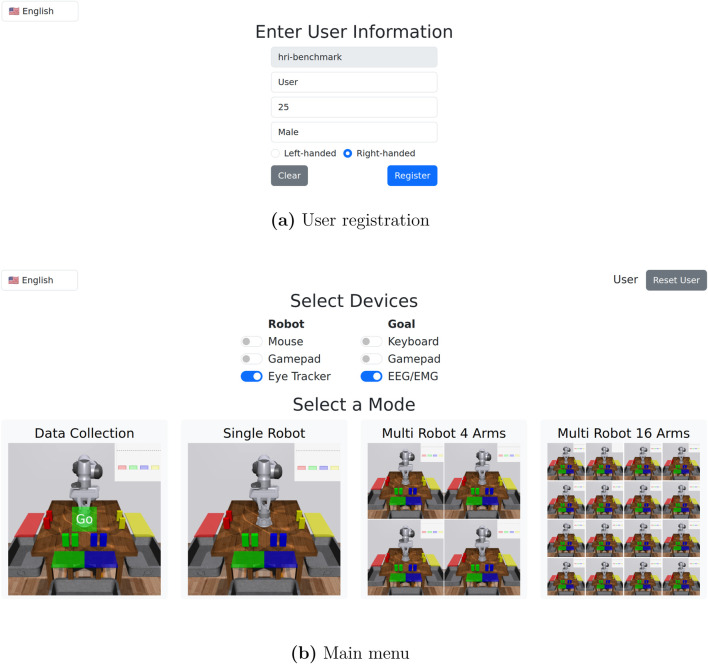
User registration and main menu. **(a)** In user registration, we collect a user name, age, gender, and handedness. **(b)** In the main menu, the user can select between different input device options, participate in data collection, or join a task execution session with either 1, 4, or 16 robots.

When first accessing the user interface via a web browser, the user is directed to a registration page (Figure 3a), where demographic information is collected. After registering, the user is directed to the main menu (Figure 3b). The user can select different input device combinations, and proceed to either data collection or task execution with differing numbers of robots.

**FIGURE 4:**
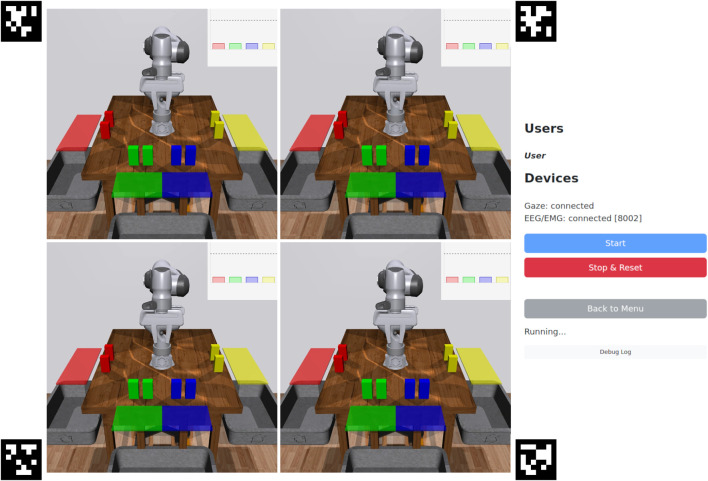
Task execution interface (4 robots). The centre contains camera views of the robots and their workspaces. The plots in the top-right display predictions over the goals, and also serves as indicators once a goal is selected. From top to bottom, the right panel contains: connected users; device connection status; start and reset experiment buttons; status info; and a collapsible debug log. If the eye tracker device is selected, AprilTags (Wang and Olson, 2016) are displayed for calibrating the eye tracker’s position with respect to the screen.

When the user enters a task execution session, they are presented with a view of the robot(s), status information, and experiment controls (Figure 4). Task execution sessions (number of robots) are shared across users, so if multiple users join the same session before it starts, they can jointly control the robot(s). If a task execution session has been started and another user tries to join, they will be blocked until the session has finished. Data collection sessions are not shared, so multiple users can collect data simultaneously.

### 3.3 Task execution and devices

During task execution, robot selection is performed by mapping a device to the cursor, and moving the cursor onto the camera view(s) (2D continuous control). For this we have implemented support for a mouse, gamepad (joystick), and a Pupil Core eye tracker. Goal (color) selection is performed by mapping a device to the four colors (4D discrete control). For this we have implemented support for a keyboard, gamepad (buttons), and g.tec EMG/EEG devices. Further devices can be added using either native browser libraries or WebSockets.

**FIGURE 5:**
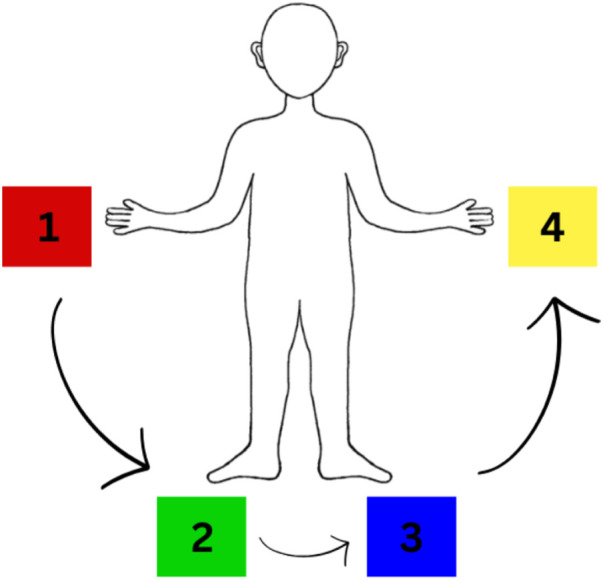
EMG mapping between muscle contractions and goal colors provided to participants during task execution.

To prevent users having to recall the color-to-goal associations in our user study (Section 4), we pasted colors on the keyboard keys (1–4), and put a diagram of the EMG mapping (Figure 5) on the wall in front of the participants. The colored gamepad buttons could directly be mapped to the goals.

Mouse and keyboard control is achieved using native browser events, and gamepad control is implemented using the native web Gamepad API.

For eye tracker control, we use the Pupil Core API^4^, which sends x-y coordinates of the user’s fixation (as well as associated confidence values) over a ZeroMQ socket^5^ to a custom device process. In our preliminary tests and pilot studies, we used the raw Pupil Core API eye tracker mappings to the screen surface, which resulted in erratic cursor movement. Based on iterative testing and users’ feedback of eye tracking stabilisation methods, we settled on averaging the last eight gaze samples with a confidence >0.75 . Finally, we send the smoothed values over WebSockets to the browser to control the cursor position.

For EMG/EEG control, we use the g.HIsys Simulink toolbox^6^ for acquiring and filtering data from g.tec devices, and stream the filtered data using Lab Streaming Layer^7^ to a custom device process. The device process can record the data, and if given a trained classifier, outputs a predicted goal, as well as a probability distribution over the goals.

### 3.4 Data collection

**FIGURE 6:**
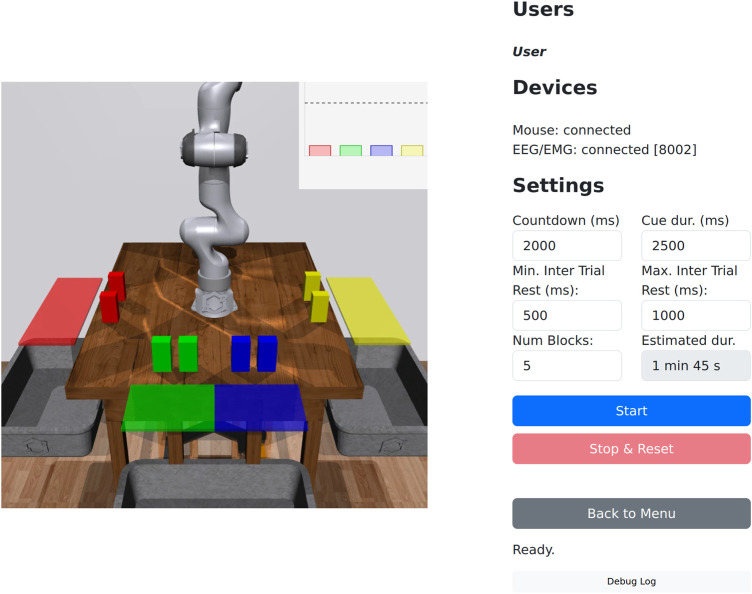
Data collection interface. A view of the robot is presented at the centre of the screen, with countdowns and cues overlaid during data collection. The experimenter can set the initial countdown, cue duration, rest periods, and the number of blocks (sets of goals).

We implemented a data collection mode that presents a randomised sequence of cues (corresponding to the different goals) to the user, and allows us to collect user input data (e.g., EMG signals) for training classification models. The duration of the cues, rest periods, number of trials, and other parameters can be set by the experimenter via the user interface (Figure 6).

In practice, we collect EMG data and train simple channel-wise threshold-based binary classifiers for each user. To map between EMG signals and the four goals (colors) in the task, we place pairs of electrodes at four sites (the left and right forearms and calves). The users are instructed on the correspondence between sites and goals, i.e., the left forearm maps to red cube, the left calf to the green cube, right calf to the blue cube, and finally the right forearm to the yellow cube. The user is then presented with a video demonstration of the data collection flow during which they practice moving the limb that corresponds to the sequentially displayed color cues until they are comfortable with their performance. The next step is to perform the actual data collection for model calibration as follows: first, a countdown of 2,000 ms is triggered, followed by a random color cue (red, green, blue or yellow) displayed for 2,500 ms during which the users contracts the appropriate limb. An inter-trial rest period of 500 ms is then introduced before proceeding to the next cue. An inter-block rest period of 1,000 ms is introduced every trial block, which is formed by four color cues. We settled for data collection with five blocks, although this can be set by the researcher (*Num Blocks* field in Figure 6) on a per-case basis. This resulted in a total of 20 trials collected over approximately 1 min and 45 s, allowing for quick experiment iterations across users.

We save the EMG data, tagged with the associated goals, in HDF5^8^, MNE FIF (Gramfort et al., 2013) and EEGLAB’s *.set* (Delorme and Makeig, 2004) formats, allowing it to be read easily by several libraries. For each site/EMG channel we train a support vector machine on the maximum signal amplitude (over a 2,500 milliseconds window) to maximise the classification accuracy. With EMG the signal’s amplitude increases as the user contracts their muscles, and thus it is straightforward to achieve high accuracies with this simple classifier. The trained classifiers are saved and deployed for the users in the following experiments.

We use Hydra (Yadan, 2019) and scikit-learn (Pedregosa et al., 2011) to automatically construct classifier pipelines from YAML files, allowing quick experimentation over different preprocessing steps and model types. For example, the aforementioned threshold classifier is specified as follows:

feature_extractor:
_target_: feature_extraction.MaximumAmplitude
vectorizer:
_target_: mne.decoding.Vectorizer
classifier:
_target_: sklearn.svm.SVC
kernel: ’poly’
C: 1
probability: True

Where the pipeline is constructed from composing each item in order, with _target_ specifying a class to instantiate, and other properties specifying the instantiation arguments.

### 3.5 Metrics

We selected a set of performance and user experience metrics that capture both task efficiency and cognitive workload. As discussed in Section 2.2, identifying metrics that are both meaningful and generalizable across HRC scenarios is a known challenge. Based on a review of commonly used metrics in the literature, we selected those that offer good adaptability across task types and experimental setups.

We evaluate system performance based on the following three quantitative metrics:

- Task Completion Time: the time from the start of the task to its successful completion. This is the most commonly used metric in HRC, as it provides a direct measure of how efficiently the human-robot team completes a given task, and offers a straightforward indicator of overall system performance. In scenarios comparing different input devices for controlling the same system, a shorter task completion time would naturally suggest a more efficient input device. Likewise, when comparing single-user and multi-user collaboration, improved coordination and division of labor in the multi-user setting would be expected to reduce the overall time required. Therefore, the lower the task completion time, the more effective the interface or interaction strategy.
- Command Selection Time: represents the time needed for the user to issue a valid command. In the single-robot scenario, it refers to the time the robot waits for a valid input. In the multi-robot case, it captures the time between selecting a robot and confirming a valid goal. This metric is crucial for evaluating the interaction process, as it reflects how quickly users can communicate their intent. It is particularly important when comparing different input modalities or control devices. A lower command selection time indicates that users can issue commands more rapidly, suggesting that the device or interface allows for efficient and fluid interaction. Therefore, systems that minimise this time are generally more intuitive and effective for user control. The metric most similar to ours in definition is the one presented in Shukla et al. (2017), where it is referred to as interaction effort or interaction time. Several studies have used similar terms, though definitions vary widely across the literature. We chose to use the term command selection time to avoid confusion about which aspect of the interaction this metric actually measures.
- Error Rate: the proportion of invalid goal selection commands sent; the command is invalid if the goal (color choice) is already completed. The entire set of valid/invalid commands are stored, so that summary statistics can be applied afterwards. This metric is essential to assess the accuracy and reliability of the human-robot interaction, as it reflects how often users attempt actions that cannot be executed, highlighting potential issues in user understanding, interface design, or system feedback. A system that enables users to make fewer errors is, by definition, more effective and better designed, as it supports more accurate and reliable interactions.

This set of quantitative metrics provides a balanced framework to evaluate key aspects of HRC. By analysing relative differences in values of these metrics, researchers can explore how different factors such as input devices, number of users, or system modifications impact overall performance and interaction quality. This approach also enables the assessment of improvements resulting from changes in system components, such as biosignal classifiers, supporting a systematic and data-driven refinement of HRI systems.

**FIGURE 7:**
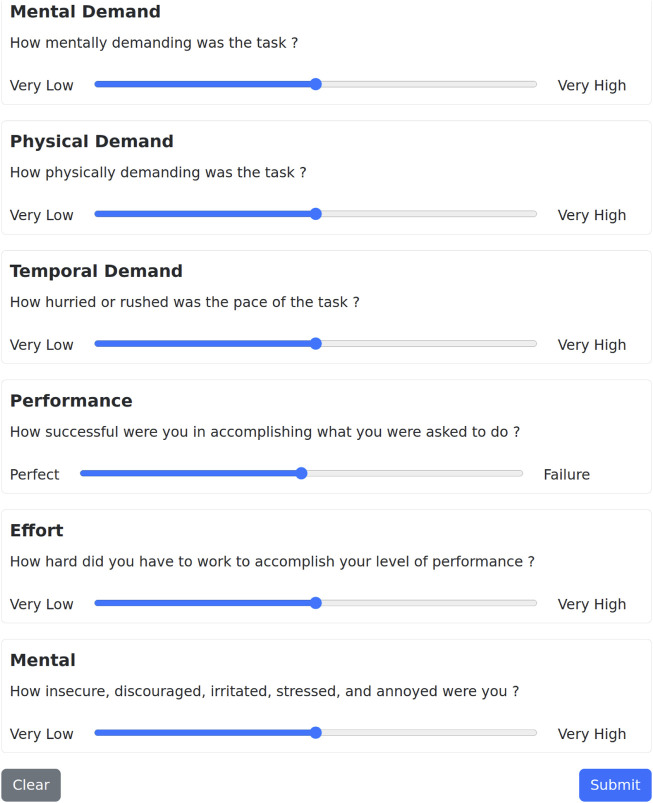
NASA TLX questionnaire. Users are presented with the six items, explanations of each item, and a slider from “Very Low” to “Very High” agreement.

In addition to quantitative performance metrics, evaluating the usability and user experience of the system is essential in human-robot interaction, where task efficiency alone does not fully capture the quality of collaboration. To this end, we selected the NASA TLX as our subjective workload assessment tool. Widely adopted in HRI studies, NASA TLX is a validated and reliable metric that captures users’ perceived cognitive and physical demands during interaction. Its multidimensional structure makes it particularly suitable for complex, interactive scenarios, such as those involving shared control between humans and robots. We created a webpage for the NASA TLX questionnaire, which users are directed to after completing an experiment (Figure 7). The questionnaire measures the user’s perceived workload over six items—Mental Demand, Physical Demand, Temporal Demand, Own Performance, Effort, and Frustration Level—using a 21-level Likert scale (normalised from 0–100, with lower values being better). The individual scores can then be averaged to calculate the overall task load index. Although it is possible to weight the items separately, we stick to the unweighted, “raw TLX” form (Hart, 2006), which provides less biased results (Bustamante and Spain, 2008).

### 3.6 Logging

We save user (demographic) data, experiment metrics, and questionnaire results in a hierarchical folder structure that resembles BIDS (Gorgolewski et al., 2016), but which separates experiment-specific and user-specific data into different folders. All of this data is stored in JSON format to be both human- and machine-readable.

### 3.7 Localisation

In order to enable users with different native languages to use our software comfortably, we implemented a language toggle (currently supporting English and Japanese), available on the user registration and main interface. This allows the experimenter/user to dynamically set the language on the user interface during experiments in order to accommodate users from different linguistic backgrounds. This feature is particularly important for collecting questionnaire data, so that questions can be conveyed in the user’s native language. The localisation code allows additional languages to be added by adding translations for relevant text to a single localisation file, where text for the interface is extracted from.

## 4 Results

### 4.1 Hypotheses

In order to demonstrate the capabilities of our benchmark, we designed and ran a user study to test several hypotheses, under two settings (Table 2). In the first setting, we investigated differences between pairs of input devices for robot and goal selection: mouse + keyboard; gamepad + gamepad; and eye tracker + EMG. In this setting, we have a single user controlling four robots, with the three different devices pairs. In the second setting, we investigated the differences between single- and multi-user control. In this setting, we have either a single user or two users control 16 robots, using the mouse + keyboard device combination. For both settings, the hypotheses we test are:

H1: There are differences in the mean task completion time.
H2: There are differences in the mean Command Selection Time.
H3: There are differences in the mean error rate.
H4: There are differences in the mean overall task load index.

**TABLE 2:** User study experimental settings. We either varied the input devices (experiment 1) or number of users (experiment 2).

Experiment	# Users	# Robots	Devices
1	1	4	mouse + keyboard vs. gamepad + gamepad vs. eye tracker + EMG
2	1 vs. 2	16	mouse + keyboard

The most effective device combination and number of users would ideally lead to a shorter task completion time (H1), a faster command selection time (H2), a lower error rate (H3), and a lower level of perceived workload (H4). Such a combination can provide a practical upper bound on performance, serving as a baseline for evaluating alternative interfaces. If another combination delivers command selection times comparable to this baseline, it can be considered functionally competitive. Lower error rates may also suggest benefits beyond accessibility, such as easier use or reduced cognitive effort.

We note that we provide these analyses as suggestions for system evaluation methods, and do not claim that one setup in necessarily superior to another. For example, our modular benchmark also allows for identifying setups tailored to individual users, accommodating diverse user needs and preferences.

### 4.2 User study

**FIGURE 8:**
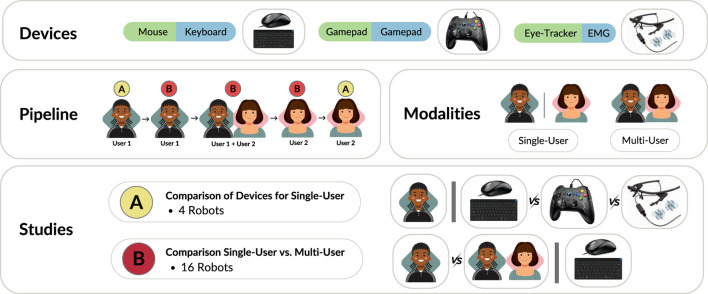
Overview of the user studies. We investigated three different combinations of devices, i.e., “Mouse and Keyboard”, “Gamepad and Gamepad”, and “Eye Tracker and EMG electrodes”. As illustrated in the pipeline, the first user performs the device comparison study with four robots, then starts the single-vs. multi-user comparison study, with the second user joining in the middle. The second user then performs the rest of their experiments in reverse order.

We recruited 50 volunteers for our user study (18 female, four left-handed, with an age distribution of 28.1 ± 7.2 years), forming 25 pairs for single-vs. multi-user control. At the beginning of the study, each user was briefed on the experiments, and asked to sign a consent form. If they consented, we proceeded with the set of experiments. Figure 8 shows the flow of the user study for pairs of users. Upon completion of the study, users were given a gift card. Our study was given ethical approval by the Shiba Palace Clinic Ethics Review Committee.

### 4.3 Input device comparison

**FIGURE 9:**
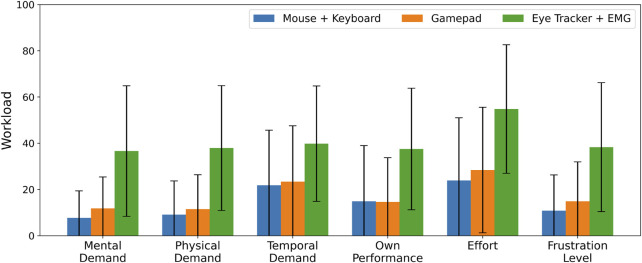
NASA TLX scores for different combinations of devices for robot and goal selection, with a single user controlling four robots. Average ± 1 standard deviation reported over 50 participants.

The overall results for the input device comparison setting are reported in Table 3 and the detailed NASA TLX results are shown in Figure 9. We checked that the data for our hypotheses was approximately Gaussian-distributed, and then ran a repeated measures ANOVA test ( α = 0.05). This test revealed significant effects of device type on Task Completion Time [F(2, 98) = 62.81, p<0.001 ], Command Selection Time [F(2, 98) = 26.74, p<0.001 ], Error Rate [F(2, 98) = 54.27, p<0.001 ], and Overall Workload [F(2, 98) = 34.66, p<0.001 ]. We then conducted *post hoc* Tukey HSD tests to examine pairwise differences. The eye tracker + EMG device pair performed significantly worse across all metrics. No significant differences were found between the mouse + keyboard and gamepad + gamepad input devices. Bonferroni correction was applied to each *p*-value from the pairwise comparisons to account for multiple comparisons (correction factor = 3 per metric, capped at 1.0):

**TABLE 3:** HRI metrics for different combinations of devices for robot and goal selection, with a single user controlling four robots. Average ± 1 standard deviation reported over 50 participants.

Robot-goal selection	Task completion time (s)	Command selection time (s)	Error rate	Overall workload
Mouse + Keyboard	105.6 ± 4.2	0.423 ± 0.443	0.005 ± 0.029	14.7 ± 13.5
Gamepad + Gamepad	106.9 ± 5.8	0.596 ± 1.155	0.014 ± 0.041	17.4 ± 15.3
Eye Tracker + EMG	132.7 ± 22.5	0.846 ± 1.284	0.292 ± 0.263	40.8 ± 20.1

Task completion time

- Eye tracker + EMG vs. gamepad + gamepad: M = −25.71; T = −9.32, **corrected** p<0.001

- Eye tracker + EMG vs. mouse + keyboard: M = −27.08; T = −9.82, **corrected** p<0.001

- Mouse + keyboard vs. gamepad + gamepad: M = 1.37; T = 0.50, corrected p = 1.0

Command selection time

- Eye tracker + EMG vs. gamepad + gamepad: M = −0.35; T = −4.98, **corrected** p<0.001

- Eye tracker + EMG vs. mouse + keyboard: M = −0.52; T = −7.47, **corrected** p<0.001

- Mouse + keyboard vs. gamepad + gamepad: M = 0.17; T = 2.49, corrected p = 0.1109

Error rate

- Eye tracker + EMG vs. gamepad + gamepad: M = −0.28; T = −8.90, **corrected** p<0.001

- Eye tracker + EMG vs. mouse + keyboard: M = −0.29; T = −9.18, **corrected** p<0.001

- Mouse + keyboard vs. gamepad + gamepad: M = 0.01; T = 0.28, corrected p = 1.0

Overall workload

- Eye tracker + EMG vs. gamepad + gamepad: M = −23.38; T = −7.00, **corrected** p<0.001

- Eye tracker + EMG vs. mouse + keyboard: M = −26.12; T = −7.82, **corrected** p<0.001

- Mouse + keyboard vs. gamepad + gamepad: M = 2.73; T = 0.82, corrected p = 1.0

The individual NASA TLX results complement this finding, as the eye tracker + EMG device pair was deemed more demanding to use across all items. Direct behavioural analysis from observing particpants also supports this, with the setup time and concentration required to perform the task with the eye tracker + EMG combination increasing the workload on the users.

### 4.4 Number of users comparison

The overall results for the number of users comparison setting are reported in Table 4 and the detailed NASA TLX results are shown in Figure 10. We checked that the data for our hypotheses was approximately Gaussian-distributed, and then ran a paired t-test ( α = 0.05). The resulting *p*-values were Bonferroni-corrected to account for multiple comparisons (correction factor = 2). No significant differences were found in Task Completion Time, Error Rate, or Overall Workload. There were no noticeable differences in the individual NASA TLX results. After correction, we found a significant difference in Command Selection Time between one and two users controlling 16 robots [t(49) = −3.71, **corrected** p=0.001 ], with two users taking longer to interact. However, because the multi-user runs always included a novice user by design, this introduces a confound, which we were able to confirm using a t-test on the difference in the mean command selection time between the first user and second user in a pair. Whilst we therefore cannot draw conclusions on H2, we leave this detail to illustrate the utility of M4Bench’s detailed metric logging.

**TABLE 4:** HRI metrics for single-vs. multi-user robot control, with users controlling 16 robots. Average ± 1 standard deviation reported over 50 participants.

# Users	Task completion time (s)	Command selection time (s)	Error rate	Overall workload
One	122.9 ± 11.7	0.237 ± 0.282	0.007 ± 0.033	22.5 ± 18.8
Two	122.6 ± 9.5	0.319 ± 0.594	0.001 ± 0.004	19.9 ± 15.7

**FIGURE 10:**
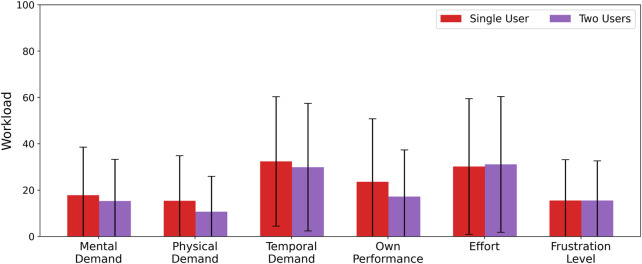
NASA TLX scores for single-vs. multi-user robot control, with users controlling 16 robots. Average ± 1 standard deviation reported over 50 participants.

### 4.5 General observations

Beyond each device’s intrinsic usability, prior experience with the devices played an important role in performance across tasks. Nearly all users were highly accustomed to keyboard and mouse setups, having used them regularly. This familiarity enabled efficient performance, with only minor differences across age groups. Conversely, elderly users were unfamiliar with gamepads, and were slightly less proficient with them. Finally, the eye tracker and EMG combination was entirely new to participants in our user study, and the brief practice sessions we conducted failed to make up for the extensive experience gap; anecdotally, the authors themselves are able to achieve similar results using all device pairs.

We also observed that the Performance item in the NASA TLX questionnaire was interpreted differently depending on the users. Some users rated themselves purely on whether the task was completed, whilst others rated themselves based on how long they took. This is of course a common issue with qualitative questionnaires.

## 5 Discussion

The flexibility of our software makes it a suitable testbed for investigating HRC with different input devices, as a precursor to more challenging tasks. For instance, this platform would allow iterating on decoding algorithms for EMG/EEG, before deploying them to real-world settings. Whilst we believe our benchmark is useful by itself, it also has greater potential for trialling novel HRI approaches that can then be ported to different scenarios.

While M4Bench is currently centred on HRC—with an emphasis on physical interaction, shared control, and collaborative manipulation—its modular and extensible architecture offers a foundation for broader applications. There are numerous improvements and additional functionalities that could be implemented in the system. For instance, adding support for new devices will enhance the platform’s flexibility, allowing it to accommodate a broader range of interfaces and enable more versatile HRI studies. Expanding customization options for device-specific parameters would make the system even more adaptable, especially for complex devices like EEG/EMG. Such devices offer a wide variety of configurations—from adjusting the number of classes to choosing paradigms and customizing training. Providing options to fine-tune these details would give researchers greater control, allowing them to optimize the system for diverse experimental needs and usage contexts.

Moreover, enhancing the platform with additional metrics would significantly improve its adaptability and relevance across diverse research contexts. This enhancement could involve adding both more system-calculated metrics and standardized qualitative measures. To further support customization, the platform could also allow researchers to pre-select the metrics most relevant to their specific study needs.

Although the platform currently calculates the metrics automatically, the analysis of the results is performed externally. A valuable enhancement would be to integrate automated analysis directly within the system. This could include the ability to compare different experimental conditions, generate detailed performance reports, and provide real-time insights, offering researchers an efficient and seamless way to evaluate their data without needing additional tools. This could improve the overall research workflow and allow for a more comprehensive understanding of the outcomes directly within the platform.

The system could include built-in basic tasks as a starting point, offering ready-made configurations for standard experimental scenarios. These basic tasks could also serve as templates, which researchers could customize further to suit their specific experimental goals.

All these features would enable researchers to tailor the M4Bench platform in detail to meet their specific objectives, making our system versatile, robust, and adaptable to a wide range of research needs and environments.

## Data Availability

The datasets presented in this article are not readily available because of privacy concerns. Requests to access the datasets should be directed to the corresponding author.

## Footnotes

1 Our investigations explained away the one significant difference found as an experimental artifact.

2
https://fastapi.tiangolo.com/

3
https://getbootstrap.com/

4
https://docs.pupil-labs.com/core/developer/network-api/

5
https://zeromq.org/

6
https://www.gtec.at/product/g-hisys/

7
https://github.com/sccn/labstreaminglayer

8
https://www.hdfgroup.org/solutions/hdf5/
